# Fusion of detected multi-channel maternal electrocardiogram (ECG) R-wave peak locations

**DOI:** 10.1186/s12938-015-0118-1

**Published:** 2016-01-08

**Authors:** Qiong Yu, Qun Guan, Ping Li, Tie-Bing Liu, Xiao-Lin Huang, Ying Zhao, Hong-Xing Liu, Yuan-Qing Wang

**Affiliations:** 1 School of Electronic Science and Engineering, Nanjing University, Xianlin Campus, Nanjing, 210023 China; 2 Nanjing General Hospital of Nanjing Military Command, Nanjing, 210002 China

**Keywords:** Multi-channel, Abdominal ECG signal, R-wave detection, Fusion

## Abstract

**Background:**

Almost all promising non-invasive foetal ECG extraction methods involve accurately determining maternal ECG R-wave peaks. However, it is not easy to robustly detect accurate R-wave peaks of the maternal ECG component in an acquired abdominal ECG since it often has a low signal-to-noise ratio (SNR), sometimes containing a large foetal ECG component or other noises and interferences. This paper discusses, under the condition of acquiring multi-channel abdominal ECG signals, how to improve the robustness of maternal ECG R-wave peak detection.

**Methods:**

On the basis of summarising the current single channel ECG R-wave peak detection methods, the paper proposed a specific fusion algorithm of detected multi-channel maternal ECG R-wave peak locations. The proposed entire algorithm was then tested using two databases; one database, created by us, was composed of 343 groups of 8-channel data collected from 78 pregnant women, and the other one, called the challenge database, was from the Physionet/Computing in Cardiology Challenge 2013, including 175 groups of 4-channel data. When using these databases, each group of data was classified into two parts, called the training part and the validation test part respectively; the training part was the first 8.192 s of each group of data and the validation test part was the next 8.192 s.

**Results:**

To show the results, three evaluation parameters—sensitivity (Se), positive predictive value (PPV) and F1—are used. The validation test results for the database we collected are Se = 99.93 %, PPV = 99.98 %, and F1 = 99.95 %, while the results for the challenge database are Se = 99.91 %, PPV = 99.86 %, and F1 = 99.88 %.

**Conclusion:**

The results of the test show that the robustness of our proposed whole fusion algorithm was superior to that of other outstanding algorithms for maternal R-wave detection, and is much better than that of single channel maternal R-wave detection algorithms.

## Background

Hospitals have strived to include foetal ECG measurements in foetal monitoring, acquiring the foetal ECG waveform and calculating the foetal heart rate. A lot of studies have been conducted to extract foetal ECG from recorded pregnant abdominal ECG signals [1–5]. However, recent revelations have indicated that the key step for a successful foetal ECG extraction is to locate the maternal ECG R-wave peaks accurately, after which, the maternal ECG component can be accurately estimated and cleanly cancelled [3–5].

It is very challenging to robustly detect accurate R-wave peaks of the maternal ECG component in an acquired abdominal ECG. Although the maternal ECG component is usually much greater than the foetal ECG component in an abdominal ECG signal, there are still some cases in which the foetal ECG component is just as great as or greater than the maternal ECG component. In these cases, a lot of incorrect maternal ECG R-wave peak detections will inevitably appear when using a single channel ECG R-wave peak detection method. This is a major obstacle for implementing foetal ECG monitoring, although it has yet to gain widespread attention.

Multi-point measurements provide an opportunity to improve the robustness of maternal ECG R-wave peak detection. One easy way is to synchronously collect one thoracic ECG signal when recording abdominal ECG signals to help locate the R-wave peaks of the maternal ECG component in recorded abdominal ECG signals [6]. However, in this method, a disadvantage is that an extra thoracic electrode is needed, which is undesirable in clinical application. On the other hand, noises and disturbances in the thoracic ECG signal sometimes will also result in incorrect R-wave peak detection when using a single channel ECG R-wave peak detection method. Alternatively, we can try to combine the acquired multi-channel abdominal ECG signals to produce a pure maternal ECG signal as a substitute for the thoracic ECG signal acquisition, based on a principal components analysis (PCA) or independent components analysis (ICA), but it is usually difficult to automatically select the pure maternal ECG signal from PCA or ICA outputs [2, 7–9].

In the Physionet/Computing in Cardiology Challenge 2013, there appear two outstanding maternal R-wave detection methods based on ICA [8, 9], called ICA$_{ACC}$ [8] and ICA$_{SMI}$ [9], respectively. The main steps of the ICA$_{ACC}$ algorithm are: (1) using FASTICA to produce N independent components for the N-channel signals, (2) initial detection on both the inputs and outputs of ICA, (3) calculating the ACC measure for each output and choosing the purest maternal ECG from the N outputs using ACCs, and (4) further processing the MQRS detections on this chosen channel using kernel density estimation, a matched filter and RR correlation to obtain more accurate MQRS detections as the final output. For the ICA$_{SMI}$ algorithm, the main steps are: (1) performing ICA on N-channel signals to obtain N independent components, (2) initial detection on both the inputs and the outputs of ICA, (3) calculating a parameter called SMI for each input and output, and (4) choosing the maternal QRS time series as the final output from the inputs and outputs using SMIs.

Noticeably, in previous research [10], an effective and simple scheme was used to improve the robustness of foetal R-wave detection after removing maternal components from the abdominal ECG signals. Its main idea is: first, to detect the foetal R-wave peaks of each estimated foetal ECG signal using a single channel ECG R-wave peak detection method, and then, to vote with the detected results of all channels’ estimated foetal ECG signals to correct the incorrect peak detections.

Referencing above, this paper will apply the new thought of fusion to the robust detection of maternal R-wave peaks of abdominal ECG signals. The proposed whole fusion algorithm consists of the first modification of the initial detection, channel selection, second modification, and voting.

The following chapters are arranged as follows. In the “Methods” chapter, first we will introduce, in detail, the two databases used for our research. Second, in the following subsections, each step of our proposed fusion algorithm will be thoroughly described and a block diagram for the algorithm will be provided. Finally, we will describe the parameter training and validation test method for the whole fusion algorithm. In the “Results” chapter, we will present the validation test results for the whole fusion algorithm, as well as for the two ICA-based maternal R-wave detection algorithms (ICA$_{ACC}$, ICA$_{SMI}$) for comparison. The final two chapters are the “Discussion” and “Conclusion”, respectively.

## Methods

**Fig. 1:**
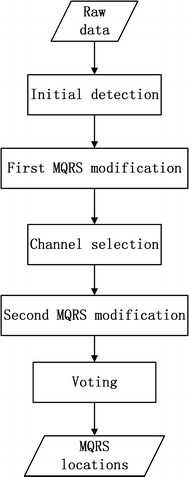
Block diagram for the entire algorithm

The block diagram for our proposed fusion algorithm can been found in Fig. 1. Our entire algorithm consists of five blocks: initial detection, first modification, channels selection, second modification and voting. In the following subsections, each individual block will be thoroughly explained.

### Data description

#### Database 1 (collected ourselves)

The authors collected actual ECG data from a hospital for our research. The methods of collecting the data can be found in the literature [11]. We conducted the collecting experiment using 78 pregnant women in the 38–40th week of gestation in the Obstetrics Branch of Nanjing General Hospital of Nanjing Military Command. The data collecting system was composed of a standard 12-lead ECG machine and a PC machine. The standard 12-lead ECG machine, made by Ni-hon Kohden Corporation with the model No. 1350p, was connected to the PC machine with a dedicated USB cable. The ECG machine acted as a data acquisition module. The parameters of this machine must be preset; the sample frequency was set to 500 Hz, the cut-off frequency of anti-aliasing filter was set to 75 Hz, and the switches for baseline drift suppression and EMG interference suppression were turned on. Previous research [11] has described the placement of the electrodes on the abdominal surface more clearly. We collected 343 groups of data in all, and every group of data was comprised of 8-channel abdominal ECG signals with a duration of 24 s. For every pregnant woman, we collected three to six groups of data.

#### Database 2 (challenge data)

The challenge data were gathered from the Physionet/Computing in Cardiology Challenge 2013. The datasets used for the challenge were obtained from five different sources (including real and simulated data), yielding a total of 447 records. All records were formatted to have a 1 kHz sampling frequency, 1-min duration, and four channels of non-invasive abdominal maternal ECG leads. The whole records were divided into three datasets for the challenge: Set A (75 records, both records and reference for FQRS locations were made public), Set B (100 records, only the records were made public), and Set C (272 records, both records and reference for FQRS locations were withheld from the public). The more detailed information about the database can be seen in the literature [12]. In our test, we only used the public datasets A and B for the test.

### Initial detection

A single channel ECG R-wave peak detection algorithm was the basis of the fusion algorithm for multi-channel ECG signals. Researchers have proposed a variety of single channel ECG R-wave peak detection algorithms, and all of these algorithms can be generalised as three steps: (1) taking a pre-processing stage for the original single channel ECG signal to improve its signal-to-noise ratio, (2) detecting the R-wave peaks on the enhanced signal obtained through the pre-processing stage, and (3) taking the initially detected R-wave peak positions of the enhanced signal as benchmarks and searching the real R-wave peaks within their neighbourhoods on the original wave. In the above three steps, the common methods for the first step are the difference method [13], the Hilbert transform method [14], the template matching method [15], the wavelet transform method [16, 17] and so on. For the second step, the common methods include all kinds of threshold setting and local searching methods [18, 19].

Among the pre-processing methods mentioned above, the robustness of the wavelet transform method was much stronger when compared to the others [20]. As long as the mother wavelet has been chosen appropriately, the wavelet transform method will suppress the baseline drift and high frequency noise at a high level. In detail, there are two ways for the wavelet transform method to enhance the original single channel ECG signal for R-wave detection: one, the modulus maxima method [21], computes the continuous wavelet transform coefficients of the original signal based on a selected wavelet and a certain scale and then takes the modulus of wavelet transform coefficients as the enhanced signal for the R-wave detection; the other was to compute the wavelet transform coefficients of the signal, followed by taking some scales’ coefficients to reconstruct an enhanced ECG signal with larger SNR [22].

This paper will apply the above first usage of the wavelet transform to do the pre-processing. In addition, before the wavelet transform, some pre-filtering methods are applied to each channel of the multi-channel data. First, a bandpass FIR filter between 0.5–80 Hz was used to suppress some noises, such as baseline wander, muscular artefacts and so on. Second, to remove the power line, two notch filters (at 50 and 60 Hz) were used. Third, to further suppress the baseline wander, a two-order smooth filter (0.2 s window) was used to obtain it and then it was subtracted from the signal.

### First modification

The first modification was used to prepare for the following channel selection, since there are usually many errors in the initial detection results and these errors will inevitably increase the difficulties in correct channel selection. The modification algorithm we used here was based on comparing each single signal’s RR interval value with the median value of all RR interval values of the channel. The main steps are as follows:

Step 1: After initial detection, for each single channel of the multi-channel signals, compute its RR interval values and mark them as *RR*s.
Step 2: Compute the median value of the *RR*s and mark it as*RR*_*m.*_
Step 3: Compute the difference value between each RR interval value and*RR*_*m*_; if the difference value is smaller than one threshold (named thres1_1; see “Results”), one of the corresponding two detected R-wave peaks needs to be removed; if the difference value is greater than one threshold (named thres1_2; see “Results”), an additional R-wave peak needs to be detected between the corresponding two detected R-wave peaks.

### Channel selection

Channel selection was an important step to guarantee the robustness of the whole fusion algorithm, since once the number of channels with erroneous single channel maternal R-wave peak detection was greater than or equal to half of the number of all channels, we cannot vote to get accurate maternal R-wave peak positions (described in next subsection).

To make the right channel selection, we used two kinds of indices to determine whether we should accept a signal. One index, named *index*1, was calculated based on the template method and the other, *index*2, was calculated using the RR interval values of the signal. The main steps of channel selection are as follows:

Step 1: After the first modification (above), re-calculate RR intervals on each channel of the multi-channel signals, then compute the median value of RR interval values of each channel and mark them as *RR*_*m*1_, *RR*_*m*2_,$\ldots$, *RR*_*mi*_, $\ldots$, *RR*_*mn*_, respectively (n refers to the number of channels of the multi-channel signals).
Step 2: Exclude the channels whose medians are lower than 0.4 s or higher than 1.6 s and mark the medians of the remaining channels as *RL*_*m*1_, *RL*_*m*2_, $\ldots$, *RL*_*mi*_, $\ldots$, *RL*_*mk*_ (*k* is the number of remaining channels).
Step 3: Compute the median of *RL*_*m*1_, *RL*_*m*2_, $\ldots$, *RL*_*mi*_, $\ldots$, *RL*_*mk*_ and mark it as *RL*_*mm*1._
Step 4: Perform further exclusions. For each remaining channel, as we previously mentioned, first compute two indices, *index*1 and *index*2. Second multiply *index*1 by *index*2 and mark the result as *index*. Third, compute the maximal value of all *index*es of the remaining channels and mark it as *ma*. Finally, compare each *index* with *ma* and if an *index* is greater than or equal to 0.5*ma, the corresponding channel is selected.

The method of calculating *index*1 of each remaining channel is as follows. For each channel’s first-modified signal, first we need to make a maternal wave complex template. In our paper, a maternal wave complex, whose duration is assigned as *RL*_*mm*1_, is defined as 0.5*RL_*mm*1_ before and 0.5*RL_*mm*1_ after the detected maternal R-wave peak location. Averaging all the maternal wave complexes synchronised on their R-wave peaks results in the maternal wave complex template. Second, compute the correlation coefficient between the template and each actual maternal wave complex. Third, compute the number of correlation coefficients that are greater than or equal to one threshold (named thres2_1; see “Results”) and then compute the ratio of this number to the number of all maternal R-wave peaks obtained by the first modification. The ratio value was taken as the *index*1of this channel.

Regarding the method of calculating *index*2, for each remaining channel, first compute the absolute difference value between its*RL*_*mi*_ and *RL*_*mm*1_, marking the absolute difference value as *med*_*diff*. Second, compute the minimum of all *med*_*diff*s of the remaining channels and mark it as *mi*. Finally, for each *med*_*diff*, if the difference value between it and*mi* is lower than one threshold (named thres2_2; see “Results”), the *index*2 of the corresponding channel is set to 0.3, and if the different value is between the thres2_2 and 2*thres2_2, the *index*2 of the corresponding channel is set to 0.2, while in other situations, the *index*2 is set to 0.1.

### Second modification

This section is also very important because it corrects the error detections on the selected channels more carefully than the first modification does, reducing the pressure to the voting and improving the robustness of the whole fusion algorithm to some degree. The second modification includes two parts: the first part is a modification using the template method and the second is a modification through the RR interval values of the signal.

The steps of the first part are as follows. First, compute the median value of all of the RR interval values of each selected channel. Then, compute the median value of all of the selected channels’ median values and mark it as *RR*_*mm*2_. Second, for each selected channel, make a maternal wave complex template whose duration is *RR*_*mm*2_ and then compute the correlation coefficient between the template and each actual maternal wave complex. Finally, remove the detected maternal R-wave peaks whose correlation coefficients are lower than one threshold (named thres3_3; see “Results”).

The fundamental idea of the second part is just like that of the first modification. It is also based on the difference values between the RR interval values and the median value of the RR intervals of each selected channel. If there is a difference value which is less than one threshold (named thres3_1; see “Results”), one of the corresponding two detected R-wave peaks will be removed; if the different value is greater than one threshold (named thres3_2; see “Results”), another R-wave peak needs to be detected between the corresponding two detected R-wave peaks.

However, there is an important detail that is different from the first modification: for different groups of data, the thresholds (thres3_1 and thres3_2) are different and are assigned according to one parameter (named *med*_*cha*) related to the actual heart rate variability (HRV) of the group of data. The method of calculating the parameter, *med*_*cha*, is described as follows:

After finishing the first part of the second modification, for each selected channel of the multi-channel signals, first, we compute its RR interval values, named *RR*s, and the median value of them, named *RR*_*median*_. Second, we exclude the RR interval values that are greater than 1.4*RR_*median*_ or less than 0.6*RR_*median*_. Third, we group the remaining RR interval values according to the original sequence and make each group consist of five values (each group can partially overlap). The number of groups can be computed using the formula *ceil*(*length*(*N*)/5), where (*N*) is the number of all remaining RR interval values of this channel. Finally, we calculated the absolute difference value between the maximal value and the minimum value of each group, and the median value of all absolute difference values for each selected channel. After finishing all of the above steps, the minimum of the median values for all selected channels was the parameter, *med*_*cha*, we needed.

Thres3_1 is set to -a*med_*cha* and thres3_2 is set to a*med_*cha* (the parameter *a* was a constant value). At the same time, the absolute value of thres3_1 and thres3_2 must be restricted to between 0.12 s and 0.25 s.

### Voting

Voting is the final step of our fusion algorithm and further corrects the error detections. Noticeably, even if the channel selection is not totally correct, we can obtain the correct results through the voting, as long as the number of error selected channels is lower than half of the number of all selected channels.

The voting part of the whole proposed fusion algorithm is based on the theory of clustering, and its basic steps are: (1) taking the detected and corrected maternal R-wave peak positions of each selected channel of the multi-channel pregnant abdominal ECG signals, (2) projecting or drawing the all detected R-peak positions of selected channels on a time axis and regarding them as samples, (3) clustering all of the samples according to their distances and labelling their classes, (4) determining whether or not the number of samples in a clustered class is greater than or equal to half of the number of all channels (if so, the class and its samples will be kept, otherwise the class and its samples will be deleted), and (5) for each remaining class, taking the median value of its samples as its representative R-wave peak position, and then searching for the peak in the neighbourhood of the representative position on each original signal, resulting in the all exact maternal R-wave peak positions of the multi-channel signals. In Step 3 above, if the distance between two samples was less than 30 ms, we assume that they belong to the same class; otherwise we assume that they belong to different classes [11]. In Step 4, we removed the classes that have few samples since we assume that they are false classes clustered with incorrectly detected peaks.

**Fig. 2:**
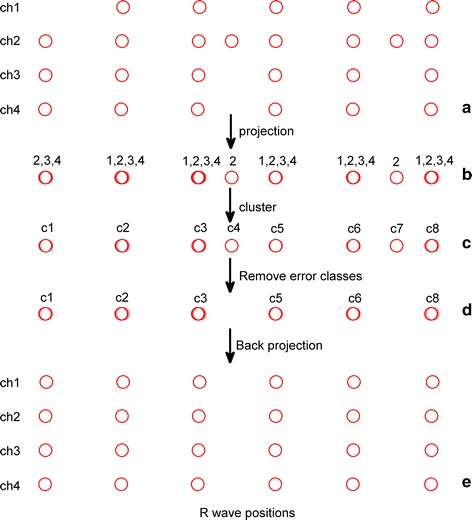
Illustration of voting. The *red circles* refer to the detected maternal R-wave peak locations. **a** The initial maternal R-wave detection results of the four-channel signals; **b** the results after projecting the initial detection results on the same *axis*; **c** cluster results for all locations; **d** remaining classes after removing *error* classes; **e** final detection results after back projecting the remaining classes to the real four-channel signals

For explicitness, the voting is illustrated in Fig. 2, taking the 4-channel signals as an example and letting red circles represent the maternal R-wave peak positions of each signal. The pseudo code of the voting is described as follows:

### Parameter training

The parameter training was used for more accurately determining the parameters’ values in our algorithm. For each group of data in each database, the first 8.192 s of the data are used as the training data. The training can be further divided into two kinds—offline training and online training. In our paper, both thres3_1 and thres3_2 training belong to online training since we must train the two parameters for each group of data and different individual data have different HRVs. Regarding the other parameters, their trainings belong to offline training, which means that once the parameters are adjusted to be good with some groups of data, the trained parameters will stay invariant for other groups of data. All of the values of the trained parameters are shown in the "Results" section.

### Validation test method

The validation test part involves three algorithms (our fusion algorithm, ICA$_{ACC)$ and ICA$_{SMI}$), as well as two above-mentioned databases (Database 1 and Database 2). Every algorithm will be tested using the two databases to compare with each other. In the validation test for each group of data in each database, only the second 8.192 s of the data are used, since we need to determine the correctness of detections through our observations and there would be a significant burden to the eyes if we used more sample points for each signal in the multi-channel signals. Additionally, though we just decided the correctness of the detections through our observations, we believe that the decisions are reliable because the data used are all multi-channel and in almost all conditions, there is at least one channel whose MQRS peaks are clearly visible.

To show the results, we use three measurement parameters—Se, PPV, and F1 [9]. The Se, PPV, and F1 are obtained by first counting the number of correctly detected maternal R-wave peaks (TP), the number of extra falsely detected maternal R-wave peaks (FP), and the number of missed maternal R-wave peaks (FN) of all groups of data used, and then calculating the three parameters’ values according to previous research [9]. In the comparison of three algorithms, the average time spent after the initial detection for each group of data will be also included.

## Results

In the test, first, we detected the maternal R-wave peaks on each signal of the multi-channel ECG signals respectively using the wavelet-based single channel R-wave detection method. The parameters of the wavelet-based method for pre-processing are set as follows: the selected mother wavelet is bior1.5 [23], the selected decomposition scale for Database 1 is 2^^^5 and for Database 2, it is 2^^^6 according to the frequency characteristics of the data (the sampling frequency).

Next, we conducted the parameter training of the fusion algorithm with Database 1, and through the training part, the parameters were finally assigned as follows: in the first modification, thres1_1 = −0.30 s and thres1_2 = 0.30 s; in the channel selection, thres2_1 = 0.6, thres2_2 = 0.06 s; in the second modification, thres3_3 = 0.5, a = 2.5. Since thres3_1 and thres3_2 are usually different for different groups of data, their values are not included in this manuscript.

After finishing the training, we conducted the test with Database 1 for our fusion algorithm. We also tested the ICA$_{ACC}$, ICA$_{SMI}$, respectively, with Database 1 to compare them to our whole fusion algorithm. The results are shown in Table 1.

**Table 1:** Results of the direct single channel maternal R-wave detection algorithm and the results of the fusion algorithm, ICA$_{ACC}$ and ICA$_{SMI}$, with Database 1

	Se (%)	PPV (%)	F1 (%)	t (s)
Initial detection	97.08	93.79	95.41	
ICA$_{ACC}$ algorithm	99.37	99.82	99.59	0.9232
ICA$_{SMI}$ algorithm	99.81	99.55	99.68	0.0073
Our fusion algorithm	99.93	99.98	99.95	0.0911

Similar to the above, next we conducted the test for the fusion algorithm and the other two algorithms with Database 2, and the results are shown in Table 2.

**Table 2:** Results of the direct single channel maternal R-wave detection algorithm and the results of the fusion algorithm, ICA$_{ACC}$ and ICA$_{SMI}$, with Database 2

	Se (%)	PPV (%)	F1 (%)	t (s)
Initial detection	94.15	89.12	91.57	
ICA$_{ACC}$ algorithm	98.93	99.41	99.17	1.7973
ICA$_{SMI}$ algorithm	99.74	98.98	99.36	0.0016
Our fusion algorithm	99.91	99.86	99.88	0.0704

**Fig. 3:**
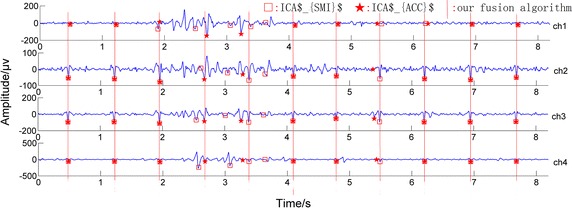
Example showing the performance of the three methods. *Filled black star* refers to the maternal R-wave peak location obtained with the ICA$_{ACC}$ algorithm, *white square* means the location obtained by the ICA$_{SMI}$ algorithm, and the *red lines* represent the locations of the R-wave peaks detected by our fusion algorithm

In addition, in Fig. 3, the record ‘b20’ in Database 2 was taken as an example to show the performance of the three methods. In the figure, ‘★’ refers to the maternal R wave peak location obtained by the ICA$_{ACC}$ algorithm, ‘□’ means the location obtained by the ICA$_{SMI}$ algorithm, and the red lines represent the locations of the R-wave peaks detected using our fusion algorithm. Observing it carefully, we can find that the fusion algorithm has detected all of the maternal R-wave peaks correctly while the other two algorithms have not.

Through the results of test for the fusion algorithm, we can see that it is a large improvement for the single channel maternal R-wave algorithms. Additionally, considering the results of the comparison test between the three algorithms, we also found that though our algorithm does not have the fastest speed, its detection results are the best and are superior to the other two algorithms.

## Discussion

**Fig. 4:**
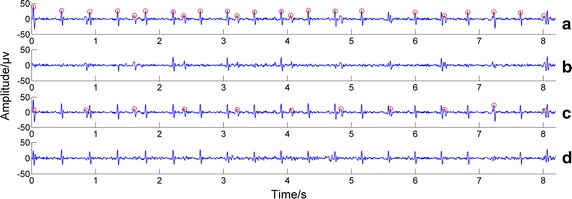
Foetal extraction results based on one single channel detection method and our fusion method. The *red circles* indicate the detected maternal R-wave peak locations. **a** The maternal ECG R-peak detection results obtained by initial single channel R-wave detection method; **b** the remaining component after maternal ECG cancelling based on the single channel R-wave detection results; **c** the maternal ECG R-peak detection results obtained by our whole fusion algorithm; **d** the remaining component after maternal ECG cancelling based on the detection results of our fusion algorithm

Our proposed fusion algorithm and other two comparative fusion algorithms are much better than single channel maternal R-wave detection algorithms. Let’s take an example to show this. The data for this example was the first channel signal of the 88th group of data in Database 1. Although the data have a low signal-to-noise ratio (containing a large foetal ECG component) and we obtained bad maternal R-wave detection results when directly using the single channel R-wave detection methods, shown in Fig. 4a, the three fusion algorithms in this paper were all able to give perfect maternal R-wave detection results, shown in Fig. 4c. Thus, with the correct maternal R-wave detection results, the ideal final foetal ECG were extracted using the foetal ECG extraction method in previous research [24], shown in Fig. 4d, which was in contrast with the bad foetal extraction result based on the single-channel R-wave detection results using the same extraction method [24], shown in Fig. 4b.

The comparative fusion algorithm ICA$_{SMI}$, involving choosing the channel with the best maternal QRS peak detection, was simple and cost the least amount of time, but it was found to be very sensitive to the initial detection, since it did not modify the initial detection results, and once the initial detection results on the selected output was incorrect due to other reasons, such as low quality, this algorithm was eventually not able to obtain the correct detection results.

**Fig. 5:**
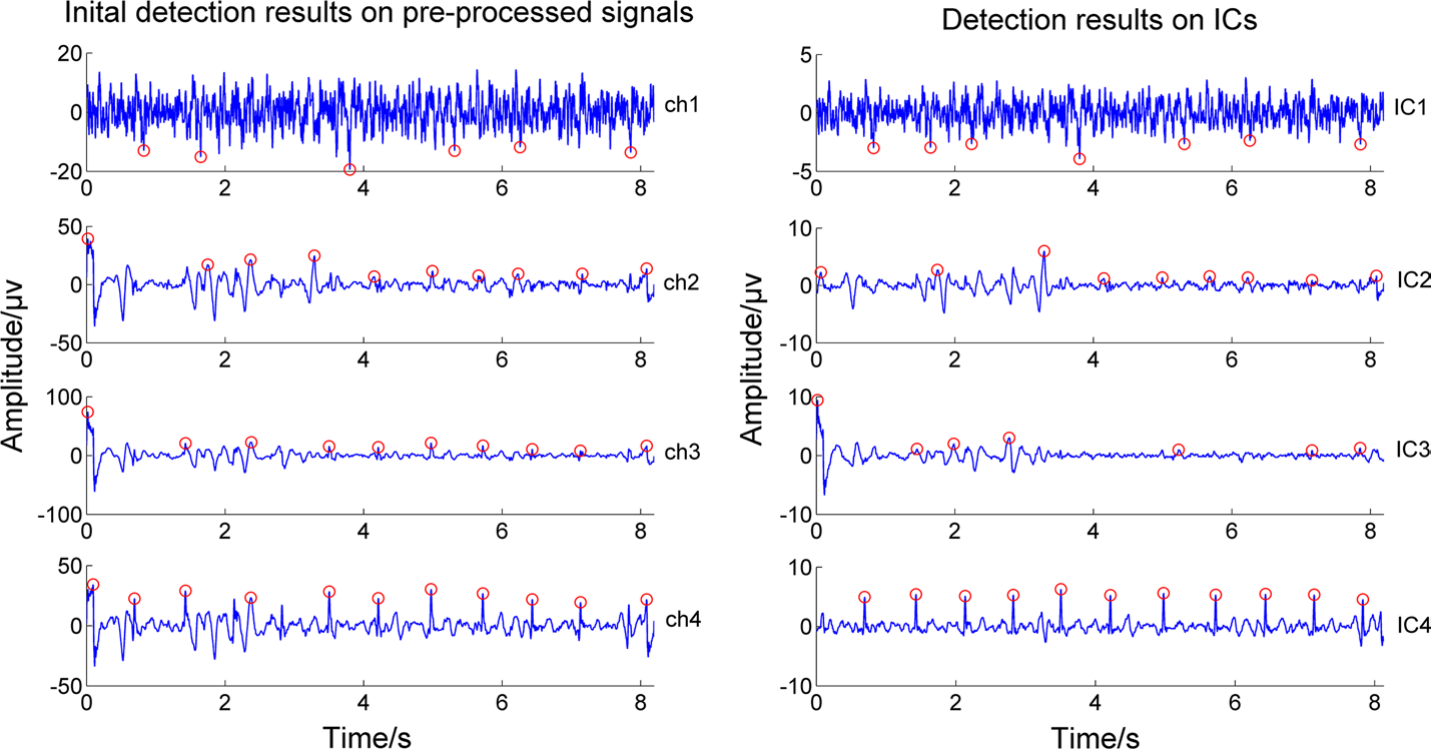
Example showing the ICA$_{ACC}$’s failure selecting for the maternal component from ICA outputs. The *red circles* refer to the detected maternal R-wave peak locations; *IC2* was selected incorrectly for its highest ACC value because the agreement between the detection results of *IC2* and the initial *ch2* was greater than the others

**Fig. 6:**
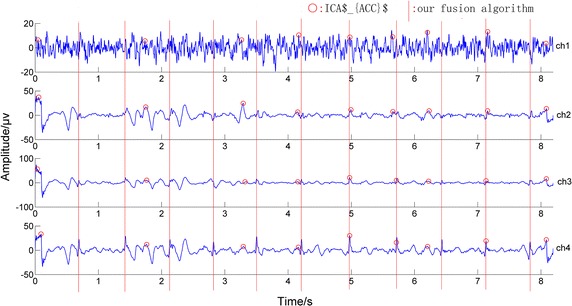
Incorrect final detection Results of ICA$_{ACC}$ due to its failure selecting for the maternal component. The *red circles* refer to the maternal R-wave peak locations detected by ICA$_{ACC}$ and the *red lines* represent the locations of the R-wave peaks detected by our fusion algorithm

Regarding another comparative fusion algorithm of ICA$_{ACC}$, although it is less sensitive to the initial detection step than ICA$_{SMI}$, it was found that a few times its channel selecting for the maternal component after doing ICA was incorrect. Let’s take the record ‘b76’ in Database 2 as an example. Both its initial detection results on pre-processed signals and the detection results on ICA outputs were shown in Fig. 5, where the red circles refer to the detected maternal R-wave peak locations. In testing, the ICA$_{ACC}$ algorithm selected the wrong maternal component (the second independent component separated, IC2), since IC2 had the highest ACC value, resulting in the incorrect final detection results, shown in Fig. 6. Nevertheless, when we applied our proposed fusion algorithm to this group of data, the correct results are obtained; in Fig. 6, the red circles refer to the maternal R-wave peak locations detected by ICA$_{ACC}$ and the red lines represent the locations of the R-wave peaks detected by our fusion algorithm. In addition to above, the fusion algorithm of ICA$_{ACC}$ was much more complex and cost the most time.

Comparing our whole fusion algorithm with other two algorithms, the time required was a little more than ICA$_{SMI}$, but much less than ICA$_{ACC}$. In comparison with the ICA$_{SMI}$, our fusion algorithm’s dependence on the initial detection was much lower since it has done a lot of work on the modification. In comparison with ICA$_{ACC}$, the voting of our fusion algorithm was slightly similar to that, but the whole fusion algorithm also had many other different elements, such as channel selection and so on. In addition, since our fusion algorithm was not based on ICA, it avoided some problems that ICA-based methods may have, such as the incorrect selection of the separated independent maternal component, and the low-quality independent component outputs of ICA due to the failed global optimal solution search when solving the ICA problem.

**Fig. 7:**
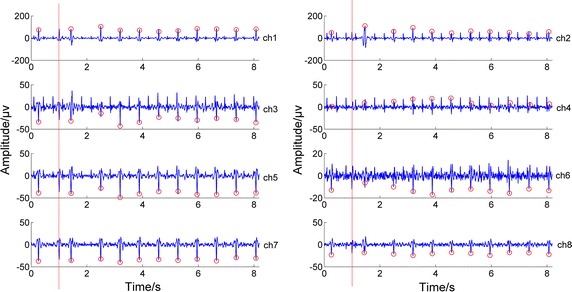
Example showing our fusion algorithm’s failure. The *red circles* indicate the detected maternal R-wave peak locations, and the *red line* represents the ignored maternal R-wave peak location

Although, in general, the proposed whole fusion algorithm was superior to others, it still did not work well for some groups of data in Databases 1 and 2. The 254th group of data in Database 1 was used as an example, shown in Fig. 7. Obviously, the second R-wave peak of each channel, which is labelled by red lines, was ignored by our algorithm, since there was a sudden HRV on the signal and the parameters of our algorithm was not able to adapt it.

Future work on our algorithm we think is how to better set the parameters of the wavelet-based single channel maternal R-wave detection algorithm, such as the selection of the mother wavelet, the selection of the decomposition scale and so on. The setting of these parameters in our paper was from previous research [23]. Perhaps they can be further optimised in the future so as to improve the initial R-wave detection results of multi-channel signals.

## Conclusion

Through the above analysis and comparison, we can conclude that though the single channel maternal R-wave detection algorithms are also useful algorithms, the detection results when directly used to detect the maternal R-wave peaks of the signal with low SNR may be not satisfactory. A lot of multi-channel maternal R wave detection algorithms based on them can greatly improve the correct rate of maternal R-wave detection in the condition of multi-channel abdominal ECG signals, especially our proposed whole fusion algorithm when compared with the other two outstanding ICA-based maternal R-wave peak detection algorithms.

In summary, the proposed complete fusion algorithm based on the single channel ECG R-wave peak detection algorithm is promising for use in improving the robustness and accuracy of the maternal R-wave detections in the condition of multi-channel abdominal ECG signals.

## Abbreviations

ECG: electrocardiogram
SNR: signal-to-noise ratio
PCA: principal components analysis
ICA: independent components analysis
HRV: heart rate variability
Se: sensitivity
PPV: positive predictive value
